# 5,5′′-Dibromo-1,1′′-bis­(prop-2-en-1-yl)-1,1′′,2,2′′-tetra­hydro­dispiro­[indole-3,7′-[6,9]diaza­tricyclo­[7.3.0.0^2,6^]dodecane-8′,3′′-indole]-2,2′′-dione

**DOI:** 10.1107/S1600536812019228

**Published:** 2012-05-05

**Authors:** Khalil Al Mamari, Hamid Ennajih, Rachid Bouhfid, El Mokhtar Essassi, Seik Weng Ng

**Affiliations:** a Laboratoire de Chimie Organique Hétérocyclique, Pôle de Compétences Pharmacochimie, Université Mohammed V–Agdal, BP 1014 Avenue Ibn Batout, Rabat, Morocco; b Institute of Nanomaterials and Nanotechnology MAScIR, Avenue de l’Armée Royale, Rabat, Morocco; c Department of Chemistry, University of Malaya, 50603 Kuala Lumpur, Malaysia; d Chemistry Department, King Abdulaziz University, PO Box 80203 Jeddah, Saudi Arabia

## Abstract

In the mol­ecule of the title compound, C_30_H_30_Br_2_N_4_O_2_, the piperazine ring adopts a chair conformation. The pyrrolidine rings that are fused to the piperazine ring adopt envelope conformations (in which the N atom represents the flap). The indoline fused-ring systems are nearly planar (r.m.s. deviations = 0.009 and 0.019 Å); the two fused rings are aligned at 60.63 (6)°.

## Related literature

For background to the class of dispiro compounds, see: Al Mamari *et al.* (2012*a* ▶). For a related structure, see: Al Mamari *et al.* (2012*b* ▶).
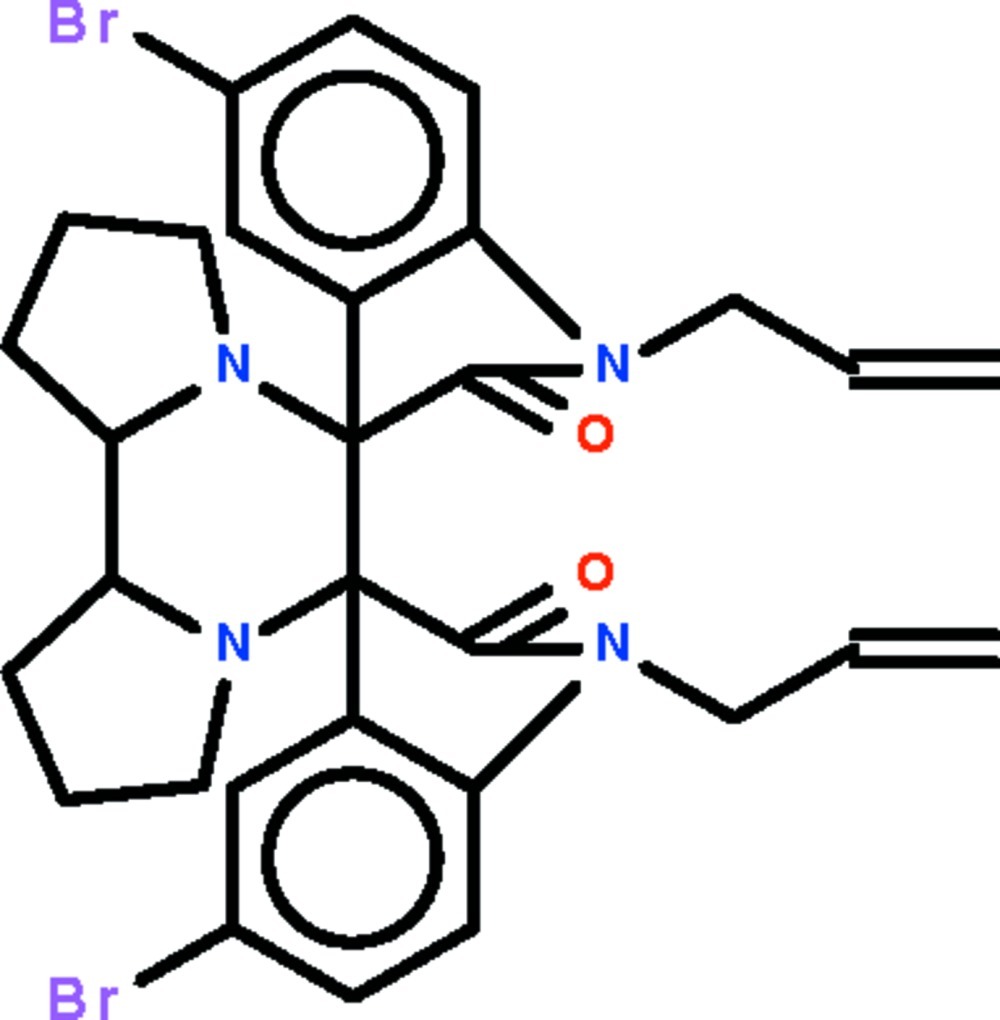


## Experimental

### 

#### Crystal data

C_30_H_30_Br_2_N_4_O_2_
*M* *_r_* = 638.40
Monoclinic, 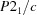

*a* = 14.1658 (3) Å
*b* = 9.7203 (2) Å
*c* = 20.6677 (3) Å
β = 100.037 (1)°
*V* = 2802.30 (9) Å^3^
*Z* = 4
Mo *K*α radiation
μ = 2.93 mm^−1^
*T* = 293 K
0.30 × 0.28 × 0.26 mm

#### Data collection

Bruker APEX DUO diffractometer
Absorption correction: multi-scan (*SADABS*; Sheldrick, 1996 ▶) *T*_min_ = 0.474, *T*_max_ = 0.517
31592 measured reflections
6671 independent reflections
4152 reflections with *I* > 2σ(*I*)
*R*_int_ = 0.050

#### Refinement

*R*[*F* ^2^ > 2σ(*F* ^2^)] = 0.040
*wR*(*F* ^2^) = 0.113
*S* = 0.96
6671 reflections
343 parameters
H-atom parameters constrained
Δρ_max_ = 0.54 e Å^−3^
Δρ_min_ = −0.61 e Å^−3^

### 

Data collection: *APEX2* (Bruker, 2010 ▶); cell refinement: *SAINT* (Bruker, 2010 ▶); data reduction: *SAINT*; program(s) used to solve structure: *SHELXS97* (Sheldrick, 2008 ▶); program(s) used to refine structure: *SHELXL97* (Sheldrick, 2008 ▶); molecular graphics: *X-SEED* (Barbour, 2001 ▶); software used to prepare material for publication: *publCIF* (Westrip, 2010 ▶).

## Supplementary Material

Crystal structure: contains datablock(s) global, I. DOI: 10.1107/S1600536812019228/xu5528sup1.cif

Structure factors: contains datablock(s) I. DOI: 10.1107/S1600536812019228/xu5528Isup2.hkl

Additional supplementary materials:  crystallographic information; 3D view; checkCIF report

## Figures and Tables

**Fig. 1.**
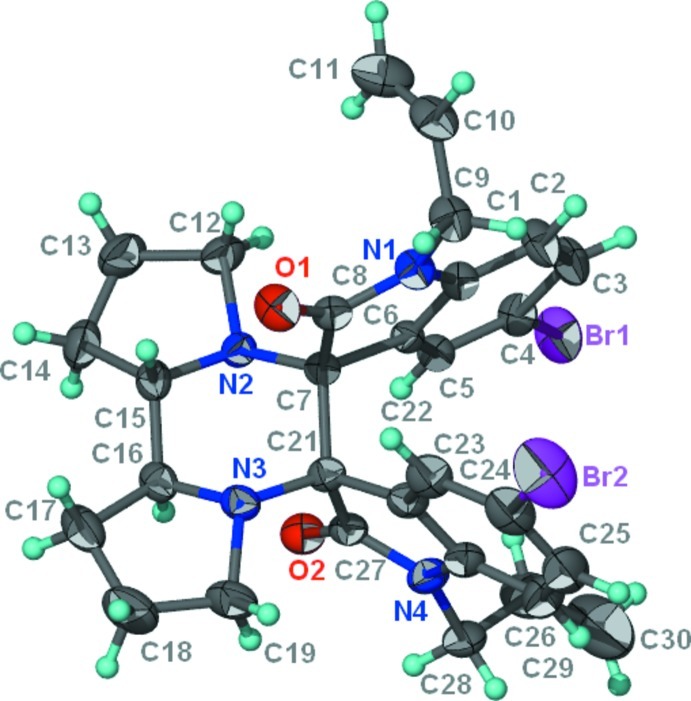
Thermal ellipsoid plot (Barbour, 2001) of C30H30Br2N4O2 at the 50% probability level.

## supplementary crystallographic information

### Comment

We reported the the 1,3-dipolar cycloaddition of 1-allyl-5-haloisatin derivatives as dipolarophiles with the azomethine ylides generated *in situ* from *N*-allylisatin and L-proline to yield dispiro-oxindoles (Al Mamari *et al.*, 2012*a*). Although one of the reactants is optically active, the product, C_30_H_30_Br_2_N_4_O_2_, crystallizes in a centrosymmetric space group, as does the bromo analog (Scheme I). However, the bromo analog is not isostructural; the two compounds have different cell dimensions. The piperazine ring adopts a chair conformation. The pyrrolidine rings that are fused to the piperazine ring adopt envelope conformations (in which the N atom represents the flap) (Fig. 1). The indoline fused-ring systems are nearly planar (r.m.s. deviation 0.009, 0.019 Å); the two fused-rings are aligned at 60.63 (6) °.

### Experimental

A mixture of 1-allyl–5-bromo-indoline-2,3-dione (1 g, 0.005 mol) and proline (0.5 g, 0.004 mole) in ethanol (20 ml) was heated for 2 hours. On completion of the reaction as indicated by TLC, water (50 ml) was added. The precipitate was collected and recrystallized from ethanol to yield colorless crystals.

### Refinement

The aromatic and methylene H-atoms were placed in calculated positions (C–H 0.93–0.97 Å) and were included in the refinement in the riding model approximation, with *U*(H) set to 1.2*U*(C).

The (0 0 2) and (0 1 1) reflections were omitted owing to bad disagreement.

### Crystal data

**Table:**

C_30_H_30_Br_2_N_4_O_2_	*F*(000) = 1296
*M**_r_* = 638.40	*D*_x_ = 1.513 Mg m^−^^3^
Monoclinic, *P*2_1_/*c*	Mo *K*α radiation, λ = 0.71073 Å
Hall symbol: -P 2ybc	Cell parameters from 8234 reflections
*a* = 14.1658 (3) Å	θ = 2.3–24.1°
*b* = 9.7203 (2) Å	µ = 2.93 mm^−^^1^
*c* = 20.6677 (3) Å	*T* = 293 K
β = 100.037 (1)°	Prism, colorless
*V* = 2802.30 (9) Å^3^	0.30 × 0.28 × 0.26 mm
*Z* = 4	

### Data collection

**Table:**

Bruker APEX DUO diffractometer	6671 independent reflections
Radiation source: fine-focus sealed tube	4152 reflections with *I* > 2σ(*I*)
Graphite monochromator	*R*_int_ = 0.050
ω scans	θ_max_ = 27.9°, θ_min_ = 2.6°
Absorption correction: multi-scan (*SADABS*; Sheldrick, 1996)	*h* = −18→17
*T*_min_ = 0.474, *T*_max_ = 0.517	*k* = −9→12
31592 measured reflections	*l* = −27→27

### Refinement

**Table:**

Refinement on *F*^2^	Primary atom site location: structure-invariant direct methods
Least-squares matrix: full	Secondary atom site location: difference Fourier map
*R*[*F*^2^ > 2σ(*F*^2^)] = 0.040	Hydrogen site location: inferred from neighbouring sites
*wR*(*F*^2^) = 0.113	H-atom parameters constrained
*S* = 0.96	*w* = 1/[σ^2^(*F*_o_^2^) + (0.0541*P*)^2^ + 1.2207*P*] where *P* = (*F*_o_^2^ + 2*F*_c_^2^)/3
6671 reflections	(Δ/σ)_max_ = 0.001
343 parameters	Δρ_max_ = 0.54 e Å^−^^3^
0 restraints	Δρ_min_ = −0.61 e Å^−^^3^

### Fractional atomic coordinates and isotropic or equivalent isotropic displacement parameters (Å^2^)

**Table:**

	*x*	*y*	*z*	*U*_iso_*/*U*_eq_	
Br1	0.58304 (2)	0.77248 (4)	0.418209 (16)	0.06146 (13)	
Br2	0.21952 (4)	0.00640 (4)	0.46489 (2)	0.09482 (18)	
O1	0.07667 (13)	0.4625 (2)	0.35513 (9)	0.0494 (5)	
O2	0.36870 (13)	0.5244 (2)	0.22067 (9)	0.0453 (5)	
C1	0.29680 (18)	0.5642 (3)	0.44015 (12)	0.0359 (6)	
C2	0.3608 (2)	0.5972 (3)	0.49586 (13)	0.0512 (8)	
H2	0.3468	0.5796	0.5374	0.061*	
C3	0.4470 (2)	0.6575 (3)	0.48829 (14)	0.0504 (7)	
H3	0.4923	0.6797	0.5250	0.061*	
C4	0.46540 (18)	0.6846 (3)	0.42628 (13)	0.0411 (6)	
C5	0.40107 (17)	0.6518 (3)	0.37021 (12)	0.0340 (6)	
H5	0.4145	0.6719	0.3288	0.041*	
C6	0.31608 (16)	0.5884 (2)	0.37742 (11)	0.0301 (5)	
C7	0.23255 (17)	0.5410 (3)	0.32613 (12)	0.0308 (5)	
C8	0.15937 (18)	0.4978 (3)	0.37166 (13)	0.0351 (6)	
C9	0.1613 (2)	0.4637 (3)	0.49103 (14)	0.0476 (7)	
H9A	0.2106	0.4221	0.5236	0.057*	
H9B	0.1142	0.3931	0.4759	0.057*	
C10	0.1140 (2)	0.5741 (4)	0.52322 (14)	0.0525 (8)	
H10	0.0930	0.5513	0.5621	0.063*	
C11	0.0991 (3)	0.6987 (4)	0.50244 (18)	0.0718 (10)	
H11A	0.1188	0.7263	0.4638	0.086*	
H11B	0.0687	0.7610	0.5261	0.086*	
C12	0.1692 (2)	0.7781 (3)	0.30457 (15)	0.0487 (7)	
H12A	0.2250	0.8309	0.3243	0.058*	
H12B	0.1281	0.7640	0.3370	0.058*	
C13	0.1150 (3)	0.8498 (4)	0.24315 (16)	0.0647 (9)	
H13A	0.0556	0.8892	0.2520	0.078*	
H13B	0.1537	0.9229	0.2294	0.078*	
C14	0.0943 (2)	0.7407 (3)	0.19024 (16)	0.0561 (8)	
H14A	0.1349	0.7528	0.1574	0.067*	
H14B	0.0277	0.7438	0.1689	0.067*	
C15	0.11682 (18)	0.6059 (3)	0.22698 (13)	0.0401 (6)	
H15	0.0620	0.5774	0.2468	0.048*	
C16	0.14914 (19)	0.4891 (3)	0.18941 (13)	0.0407 (6)	
H16	0.2029	0.5189	0.1687	0.049*	
C17	0.0744 (2)	0.4164 (4)	0.13854 (15)	0.0578 (8)	
H17A	0.0115	0.4209	0.1509	0.069*	
H17B	0.0712	0.4578	0.0955	0.069*	
C18	0.1094 (3)	0.2683 (4)	0.13855 (19)	0.0803 (12)	
H18A	0.0586	0.2052	0.1446	0.096*	
H18B	0.1298	0.2469	0.0973	0.096*	
C19	0.1929 (2)	0.2564 (3)	0.19506 (16)	0.0566 (8)	
H19A	0.1897	0.1714	0.2192	0.068*	
H19B	0.2537	0.2603	0.1795	0.068*	
C20	0.35499 (18)	0.4387 (3)	0.26056 (13)	0.0356 (6)	
C21	0.26015 (17)	0.4105 (2)	0.28799 (12)	0.0339 (6)	
C22	0.29012 (19)	0.2894 (3)	0.33298 (13)	0.0398 (6)	
C23	0.2387 (2)	0.2126 (3)	0.37111 (15)	0.0494 (7)	
H23	0.1746	0.2315	0.3721	0.059*	
C24	0.2863 (3)	0.1059 (3)	0.40796 (15)	0.0590 (9)	
C25	0.3796 (3)	0.0721 (3)	0.40497 (17)	0.0662 (10)	
H25	0.4086	−0.0010	0.4298	0.079*	
C26	0.4307 (2)	0.1456 (3)	0.36554 (16)	0.0586 (8)	
H26	0.4936	0.1226	0.3627	0.070*	
C27	0.3849 (2)	0.2546 (3)	0.33049 (14)	0.0428 (7)	
C28	0.5159 (2)	0.3350 (4)	0.26852 (15)	0.0544 (8)	
H28A	0.5108	0.3652	0.2233	0.065*	
H28B	0.5363	0.2396	0.2706	0.065*	
C29	0.5904 (2)	0.4189 (5)	0.31056 (19)	0.0817 (12)	
H29	0.5719	0.5061	0.3220	0.098*	
C30	0.6727 (3)	0.3861 (7)	0.3315 (2)	0.133 (2)	
H30A	0.6950	0.3001	0.3215	0.159*	
H30B	0.7135	0.4470	0.3575	0.159*	
N1	0.20466 (15)	0.5078 (2)	0.43570 (11)	0.0387 (5)	
N2	0.19727 (14)	0.6467 (2)	0.27880 (10)	0.0334 (5)	
N3	0.18063 (14)	0.3764 (2)	0.23592 (10)	0.0381 (5)	
N4	0.42146 (16)	0.3437 (2)	0.28745 (11)	0.0430 (5)	

### Atomic displacement parameters (Å^2^)

**Table:**

	*U*^11^	*U*^22^	*U*^33^	*U*^12^	*U*^13^	*U*^23^
Br1	0.03869 (17)	0.0799 (3)	0.0637 (2)	−0.02284 (15)	0.00310 (14)	−0.00209 (17)
Br2	0.1388 (4)	0.0657 (3)	0.0792 (3)	−0.0361 (2)	0.0167 (3)	0.0224 (2)
O1	0.0349 (10)	0.0619 (13)	0.0521 (12)	−0.0160 (9)	0.0098 (9)	−0.0044 (10)
O2	0.0421 (11)	0.0487 (12)	0.0479 (11)	−0.0025 (9)	0.0156 (9)	0.0003 (10)
C1	0.0338 (13)	0.0370 (15)	0.0373 (14)	−0.0030 (11)	0.0075 (11)	0.0020 (12)
C2	0.0512 (17)	0.070 (2)	0.0319 (14)	−0.0084 (15)	0.0054 (13)	0.0032 (14)
C3	0.0422 (16)	0.067 (2)	0.0381 (15)	−0.0104 (15)	−0.0034 (12)	−0.0031 (14)
C4	0.0318 (13)	0.0437 (16)	0.0468 (16)	−0.0067 (12)	0.0044 (12)	−0.0001 (13)
C5	0.0330 (13)	0.0355 (14)	0.0345 (13)	−0.0044 (11)	0.0087 (11)	0.0004 (11)
C6	0.0302 (12)	0.0298 (13)	0.0301 (12)	−0.0003 (10)	0.0048 (10)	−0.0022 (10)
C7	0.0290 (12)	0.0313 (13)	0.0330 (13)	−0.0028 (10)	0.0079 (10)	−0.0017 (11)
C8	0.0348 (14)	0.0328 (14)	0.0395 (14)	−0.0021 (11)	0.0113 (11)	−0.0021 (11)
C9	0.0464 (16)	0.0577 (19)	0.0410 (15)	−0.0092 (14)	0.0146 (13)	0.0076 (14)
C10	0.0531 (18)	0.067 (2)	0.0412 (16)	−0.0120 (16)	0.0201 (14)	−0.0020 (16)
C11	0.087 (3)	0.069 (3)	0.067 (2)	−0.007 (2)	0.037 (2)	−0.013 (2)
C12	0.0523 (17)	0.0370 (16)	0.0568 (18)	0.0086 (13)	0.0093 (14)	−0.0029 (14)
C13	0.081 (2)	0.053 (2)	0.062 (2)	0.0252 (18)	0.0178 (18)	0.0126 (17)
C14	0.0487 (17)	0.062 (2)	0.0545 (17)	0.0136 (15)	0.0004 (14)	0.0120 (16)
C15	0.0307 (13)	0.0494 (17)	0.0388 (14)	−0.0001 (12)	0.0020 (11)	0.0009 (13)
C16	0.0347 (14)	0.0507 (17)	0.0364 (14)	−0.0047 (12)	0.0057 (11)	−0.0035 (13)
C17	0.0502 (18)	0.080 (2)	0.0410 (16)	−0.0097 (17)	0.0022 (13)	−0.0119 (16)
C18	0.082 (3)	0.084 (3)	0.070 (2)	−0.011 (2)	−0.003 (2)	−0.039 (2)
C19	0.062 (2)	0.0503 (19)	0.0578 (19)	−0.0069 (15)	0.0118 (16)	−0.0219 (15)
C20	0.0343 (14)	0.0347 (15)	0.0384 (14)	−0.0005 (12)	0.0076 (11)	−0.0086 (12)
C21	0.0325 (13)	0.0300 (14)	0.0396 (14)	−0.0025 (11)	0.0071 (11)	−0.0034 (11)
C22	0.0470 (15)	0.0289 (14)	0.0437 (15)	−0.0013 (12)	0.0082 (12)	−0.0053 (12)
C23	0.0611 (19)	0.0333 (16)	0.0532 (17)	−0.0076 (14)	0.0086 (15)	−0.0010 (14)
C24	0.088 (3)	0.0355 (17)	0.0518 (18)	−0.0125 (17)	0.0087 (17)	0.0008 (14)
C25	0.098 (3)	0.0369 (19)	0.058 (2)	0.0124 (18)	−0.002 (2)	0.0019 (16)
C26	0.067 (2)	0.0466 (19)	0.0586 (19)	0.0157 (16)	−0.0003 (16)	−0.0007 (16)
C27	0.0491 (16)	0.0332 (15)	0.0447 (15)	0.0044 (12)	0.0040 (13)	−0.0086 (12)
C28	0.0423 (16)	0.071 (2)	0.0512 (17)	0.0166 (15)	0.0115 (14)	−0.0123 (16)
C29	0.043 (2)	0.123 (4)	0.081 (3)	0.004 (2)	0.0170 (18)	−0.035 (2)
C30	0.058 (3)	0.243 (7)	0.099 (3)	−0.011 (4)	0.018 (2)	−0.057 (4)
N1	0.0345 (12)	0.0471 (14)	0.0368 (12)	−0.0066 (10)	0.0124 (9)	0.0015 (10)
N2	0.0336 (11)	0.0312 (12)	0.0352 (11)	0.0019 (9)	0.0049 (9)	0.0003 (9)
N3	0.0362 (12)	0.0382 (13)	0.0394 (12)	−0.0048 (10)	0.0049 (10)	−0.0095 (10)
N4	0.0379 (12)	0.0431 (14)	0.0490 (13)	0.0069 (11)	0.0106 (10)	−0.0055 (11)

### Geometric parameters (Å, º)

**Table:**

Br1—C4	1.906 (3)	C15—N2	1.476 (3)
Br2—C24	1.898 (3)	C15—C16	1.492 (4)
O1—C8	1.212 (3)	C15—H15	0.9800
O2—C20	1.211 (3)	C16—N3	1.474 (3)
C1—C2	1.374 (4)	C16—C17	1.528 (4)
C1—C6	1.391 (3)	C16—H16	0.9800
C1—N1	1.404 (3)	C17—C18	1.523 (5)
C2—C3	1.388 (4)	C17—H17A	0.9700
C2—H2	0.9300	C17—H17B	0.9700
C3—C4	1.377 (4)	C18—C19	1.515 (5)
C3—H3	0.9300	C18—H18A	0.9700
C4—C5	1.381 (3)	C18—H18B	0.9700
C5—C6	1.384 (3)	C19—N3	1.468 (3)
C5—H5	0.9300	C19—H19A	0.9700
C6—C7	1.516 (3)	C19—H19B	0.9700
C7—N2	1.446 (3)	C20—N4	1.365 (3)
C7—C8	1.574 (3)	C20—C21	1.571 (3)
C7—C21	1.578 (3)	C21—N3	1.454 (3)
C8—N1	1.370 (3)	C21—C22	1.514 (4)
C9—N1	1.454 (3)	C22—C23	1.382 (4)
C9—C10	1.482 (4)	C22—C27	1.394 (4)
C9—H9A	0.9700	C23—C24	1.390 (4)
C9—H9B	0.9700	C23—H23	0.9300
C10—C11	1.291 (5)	C24—C25	1.373 (5)
C10—H10	0.9300	C25—C26	1.380 (5)
C11—H11A	0.9300	C25—H25	0.9300
C11—H11B	0.9300	C26—C27	1.380 (4)
C12—N2	1.466 (3)	C26—H26	0.9300
C12—C13	1.532 (4)	C27—N4	1.404 (4)
C12—H12A	0.9700	C28—N4	1.461 (3)
C12—H12B	0.9700	C28—C29	1.488 (5)
C13—C14	1.515 (5)	C28—H28A	0.9700
C13—H13A	0.9700	C28—H28B	0.9700
C13—H13B	0.9700	C29—C30	1.214 (6)
C14—C15	1.520 (4)	C29—H29	0.9300
C14—H14A	0.9700	C30—H30A	0.9300
C14—H14B	0.9700	C30—H30B	0.9300
			
C2—C1—C6	122.3 (2)	C18—C17—C16	104.5 (3)
C2—C1—N1	128.0 (2)	C18—C17—H17A	110.8
C6—C1—N1	109.7 (2)	C16—C17—H17A	110.8
C1—C2—C3	118.0 (3)	C18—C17—H17B	110.8
C1—C2—H2	121.0	C16—C17—H17B	110.8
C3—C2—H2	121.0	H17A—C17—H17B	108.9
C4—C3—C2	119.9 (3)	C19—C18—C17	106.4 (3)
C4—C3—H3	120.1	C19—C18—H18A	110.4
C2—C3—H3	120.1	C17—C18—H18A	110.4
C3—C4—C5	122.2 (2)	C19—C18—H18B	110.4
C3—C4—Br1	118.5 (2)	C17—C18—H18B	110.4
C5—C4—Br1	119.3 (2)	H18A—C18—H18B	108.6
C4—C5—C6	118.1 (2)	N3—C19—C18	103.2 (3)
C4—C5—H5	120.9	N3—C19—H19A	111.1
C6—C5—H5	120.9	C18—C19—H19A	111.1
C5—C6—C1	119.5 (2)	N3—C19—H19B	111.1
C5—C6—C7	130.4 (2)	C18—C19—H19B	111.1
C1—C6—C7	110.1 (2)	H19A—C19—H19B	109.1
N2—C7—C6	113.1 (2)	O2—C20—N4	124.1 (2)
N2—C7—C8	114.1 (2)	O2—C20—C21	127.9 (2)
C6—C7—C8	100.27 (19)	N4—C20—C21	107.9 (2)
N2—C7—C21	108.64 (19)	N3—C21—C22	112.1 (2)
C6—C7—C21	111.26 (19)	N3—C21—C20	111.9 (2)
C8—C7—C21	109.31 (19)	C22—C21—C20	101.0 (2)
O1—C8—N1	124.0 (2)	N3—C21—C7	108.97 (19)
O1—C8—C7	127.8 (2)	C22—C21—C7	112.7 (2)
N1—C8—C7	108.2 (2)	C20—C21—C7	109.99 (19)
N1—C9—C10	115.2 (3)	C23—C22—C27	119.8 (3)
N1—C9—H9A	108.5	C23—C22—C21	130.6 (3)
C10—C9—H9A	108.5	C27—C22—C21	109.5 (2)
N1—C9—H9B	108.5	C22—C23—C24	117.5 (3)
C10—C9—H9B	108.5	C22—C23—H23	121.3
H9A—C9—H9B	107.5	C24—C23—H23	121.3
C11—C10—C9	126.2 (3)	C25—C24—C23	122.2 (3)
C11—C10—H10	116.9	C25—C24—Br2	119.6 (3)
C9—C10—H10	116.9	C23—C24—Br2	118.2 (3)
C10—C11—H11A	120.0	C24—C25—C26	120.7 (3)
C10—C11—H11B	120.0	C24—C25—H25	119.6
H11A—C11—H11B	120.0	C26—C25—H25	119.6
N2—C12—C13	102.9 (2)	C25—C26—C27	117.4 (3)
N2—C12—H12A	111.2	C25—C26—H26	121.3
C13—C12—H12A	111.2	C27—C26—H26	121.3
N2—C12—H12B	111.2	C26—C27—C22	122.4 (3)
C13—C12—H12B	111.2	C26—C27—N4	127.9 (3)
H12A—C12—H12B	109.1	C22—C27—N4	109.7 (2)
C14—C13—C12	106.6 (2)	N4—C28—C29	113.7 (2)
C14—C13—H13A	110.4	N4—C28—H28A	108.8
C12—C13—H13A	110.4	C29—C28—H28A	108.8
C14—C13—H13B	110.4	N4—C28—H28B	108.8
C12—C13—H13B	110.4	C29—C28—H28B	108.8
H13A—C13—H13B	108.6	H28A—C28—H28B	107.7
C13—C14—C15	104.2 (2)	C30—C29—C28	127.1 (5)
C13—C14—H14A	110.9	C30—C29—H29	116.4
C15—C14—H14A	110.9	C28—C29—H29	116.4
C13—C14—H14B	110.9	C29—C30—H30A	120.0
C15—C14—H14B	110.9	C29—C30—H30B	120.0
H14A—C14—H14B	108.9	H30A—C30—H30B	120.0
N2—C15—C16	108.3 (2)	C8—N1—C1	111.4 (2)
N2—C15—C14	101.5 (2)	C8—N1—C9	123.2 (2)
C16—C15—C14	116.9 (2)	C1—N1—C9	125.4 (2)
N2—C15—H15	109.9	C7—N2—C12	117.2 (2)
C16—C15—H15	109.9	C7—N2—C15	115.6 (2)
C14—C15—H15	109.9	C12—N2—C15	105.9 (2)
N3—C16—C15	108.1 (2)	C21—N3—C19	116.9 (2)
N3—C16—C17	101.5 (2)	C21—N3—C16	114.9 (2)
C15—C16—C17	118.1 (2)	C19—N3—C16	105.5 (2)
N3—C16—H16	109.5	C20—N4—C27	111.7 (2)
C15—C16—H16	109.5	C20—N4—C28	122.0 (2)
C17—C16—H16	109.5	C27—N4—C28	126.2 (2)
			
C6—C1—C2—C3	0.4 (4)	C27—C22—C23—C24	2.7 (4)
N1—C1—C2—C3	−177.4 (3)	C21—C22—C23—C24	−179.9 (3)
C1—C2—C3—C4	1.1 (5)	C22—C23—C24—C25	−3.0 (5)
C2—C3—C4—C5	−1.0 (5)	C22—C23—C24—Br2	176.1 (2)
C2—C3—C4—Br1	178.2 (2)	C23—C24—C25—C26	1.2 (5)
C3—C4—C5—C6	−0.7 (4)	Br2—C24—C25—C26	−178.0 (2)
Br1—C4—C5—C6	−179.82 (19)	C24—C25—C26—C27	1.0 (5)
C4—C5—C6—C1	2.2 (4)	C25—C26—C27—C22	−1.2 (4)
C4—C5—C6—C7	179.8 (2)	C25—C26—C27—N4	−179.9 (3)
C2—C1—C6—C5	−2.1 (4)	C23—C22—C27—C26	−0.6 (4)
N1—C1—C6—C5	176.1 (2)	C21—C22—C27—C26	−178.5 (3)
C2—C1—C6—C7	179.8 (3)	C23—C22—C27—N4	178.3 (2)
N1—C1—C6—C7	−2.0 (3)	C21—C22—C27—N4	0.4 (3)
C5—C6—C7—N2	−51.2 (3)	N4—C28—C29—C30	138.3 (5)
C1—C6—C7—N2	126.6 (2)	O1—C8—N1—C1	−174.0 (2)
C5—C6—C7—C8	−173.1 (3)	C7—C8—N1—C1	5.4 (3)
C1—C6—C7—C8	4.7 (3)	O1—C8—N1—C9	6.4 (4)
C5—C6—C7—C21	71.4 (3)	C7—C8—N1—C9	−174.2 (2)
C1—C6—C7—C21	−110.8 (2)	C2—C1—N1—C8	175.8 (3)
N2—C7—C8—O1	52.2 (3)	C6—C1—N1—C8	−2.3 (3)
C6—C7—C8—O1	173.4 (3)	C2—C1—N1—C9	−4.6 (4)
C21—C7—C8—O1	−69.6 (3)	C6—C1—N1—C9	177.3 (2)
N2—C7—C8—N1	−127.2 (2)	C10—C9—N1—C8	−96.0 (3)
C6—C7—C8—N1	−6.0 (3)	C10—C9—N1—C1	84.5 (3)
C21—C7—C8—N1	111.0 (2)	C6—C7—N2—C12	−54.9 (3)
N1—C9—C10—C11	8.1 (5)	C8—C7—N2—C12	58.9 (3)
N2—C12—C13—C14	14.0 (3)	C21—C7—N2—C12	−178.9 (2)
C12—C13—C14—C15	12.4 (3)	C6—C7—N2—C15	179.1 (2)
C13—C14—C15—N2	−33.9 (3)	C8—C7—N2—C15	−67.1 (3)
C13—C14—C15—C16	−151.5 (3)	C21—C7—N2—C15	55.1 (2)
N2—C15—C16—N3	57.9 (3)	C13—C12—N2—C7	−167.2 (2)
C14—C15—C16—N3	171.7 (2)	C13—C12—N2—C15	−36.6 (3)
N2—C15—C16—C17	172.4 (2)	C16—C15—N2—C7	−60.2 (3)
C14—C15—C16—C17	−73.9 (3)	C14—C15—N2—C7	176.2 (2)
N3—C16—C17—C18	−30.9 (3)	C16—C15—N2—C12	168.2 (2)
C15—C16—C17—C18	−148.9 (3)	C14—C15—N2—C12	44.6 (3)
C16—C17—C18—C19	7.9 (4)	C22—C21—N3—C19	−54.2 (3)
C17—C18—C19—N3	18.3 (4)	C20—C21—N3—C19	58.5 (3)
O2—C20—C21—N3	58.6 (3)	C7—C21—N3—C19	−179.6 (2)
N4—C20—C21—N3	−118.6 (2)	C22—C21—N3—C16	−178.7 (2)
O2—C20—C21—C22	178.0 (3)	C20—C21—N3—C16	−66.0 (3)
N4—C20—C21—C22	0.8 (2)	C7—C21—N3—C16	55.9 (3)
O2—C20—C21—C7	−62.7 (3)	C18—C19—N3—C21	−168.3 (3)
N4—C20—C21—C7	120.1 (2)	C18—C19—N3—C16	−39.2 (3)
N2—C7—C21—N3	−50.1 (2)	C15—C16—N3—C21	−60.8 (3)
C6—C7—C21—N3	−175.23 (19)	C17—C16—N3—C21	174.3 (2)
C8—C7—C21—N3	75.0 (2)	C15—C16—N3—C19	169.0 (2)
N2—C7—C21—C22	−175.15 (19)	C17—C16—N3—C19	44.0 (3)
C6—C7—C21—C22	59.7 (3)	O2—C20—N4—C27	−178.0 (2)
C8—C7—C21—C22	−50.1 (3)	C21—C20—N4—C27	−0.6 (3)
N2—C7—C21—C20	73.0 (2)	O2—C20—N4—C28	−1.8 (4)
C6—C7—C21—C20	−52.2 (3)	C21—C20—N4—C28	175.6 (2)
C8—C7—C21—C20	−162.0 (2)	C26—C27—N4—C20	179.0 (3)
N3—C21—C22—C23	−59.0 (4)	C22—C27—N4—C20	0.2 (3)
C20—C21—C22—C23	−178.3 (3)	C26—C27—N4—C28	3.0 (4)
C7—C21—C22—C23	64.4 (4)	C22—C27—N4—C28	−175.8 (2)
N3—C21—C22—C27	118.6 (2)	C29—C28—N4—C20	91.7 (4)
C20—C21—C22—C27	−0.7 (3)	C29—C28—N4—C27	−92.6 (4)
C7—C21—C22—C27	−118.0 (2)		
